# COVID-19 pneumonia detected by [^18^F]FDG PET/MRI: a case with negative antigen test and chest X-ray results

**DOI:** 10.1259/bjrcr.20210131

**Published:** 2022-03-09

**Authors:** Tetsuya Tsujikawa, Masaki Anzai, Yukihiro Umeda, Hideaki Tsuyoshi, Nobuyuki Kosaka, Hirohiko Kimura, Hidehiko Okazawa

**Affiliations:** 1 Biomedical Imaging Research Center, University of Fukui, Fukui, Japan; 2 Third Department of Internal Medicine, Faculty of Medical Sciences, University of Fukui, Fukui, Japan; 3 Department of Obstetrics and Gynecology, Faculty of Medical Sciences, University of Fukui, Fukui, Japan; 4 Department of Radiology, Faculty of Medical Sciences, University of Fukui, Fukui, Japan

## Abstract

Since the outbreak of pneumonia caused by a novel coronavirus (SARS-CoV-2) named Coronavirus disease 2019 (COVID-19) in China, researchers have reported the fluorodeoxyglucose positron emission tomography/CT (FDG PET/CT) manifestations of COVID-19 infection. We present a 37-year-old female with early-stage cervical cancer and fever without a focus who had negative SARS-CoV-2 antigen test and chest X-ray results. FDG PET/MRI performed for preoperative evaluation incidentally detected pneumonia showing high FDG uptake and diffusion-weighted imaging signals in right lung base. She retested positive for SARS-CoV-2 and was diagnosed as having COVID-19 pneumonia. Whole-body PET/MRI can provide multi functional images and could be useful for evaluating the pathophysiology of COVID-19.

## Case presentation

**Figure 1.**
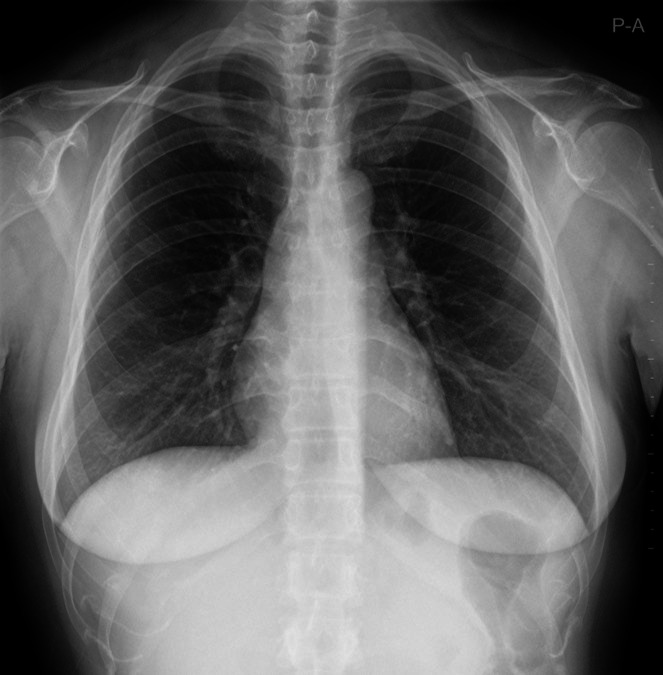
Chest X-ray was normal.

**Figure 2.**
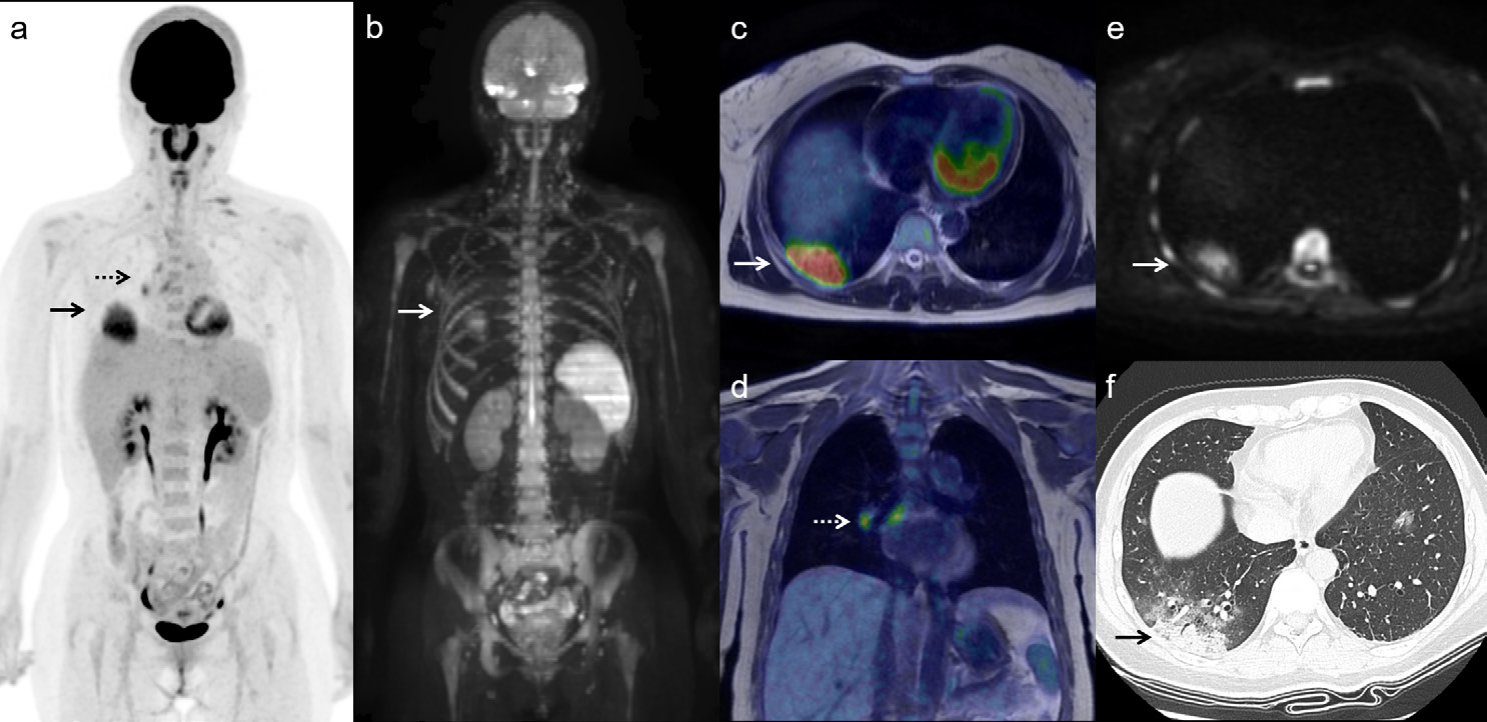
Maximal intensity projection images of FDG PET (**a**) and DWI (**b**), transaxial and coronal FDG PET/*T*_2_ weighted MR fusion images (**c and d**) and transaxial DWI (**e**) show an FDG-avid and high-intense pulmonary lesion (arrows) and FDG-avid ipsilateral hilar and mediastinal nodes (dotted arrows). High-resolution CT performed 2 days later (**f**) shows consolidation in right lung base periphery surrounded by ground-glass opacities consistent with COVID-19 pneumonia (arrow). DWI, diffusion-weighted imaging; FDG, fluorodeoxyglucose; PET, positron emission tomography

We present herein a 37-year-old female with early-stage cervical cancer and fever without a focus lasting for 5 days. She tested negative for SARS-CoV-2 antigen and the chest X-ray was normal (Figure 1). She subsequently underwent a fluorodeoxyglucose positron emission tomography/MRI (FDG PET/MRI) scan for pre-operative evaluation of cervical cancer wearing an MRI-safe face mask with no metal. PET/MRI incidentally detected pneumonia showing high FDG uptake (Figure 2a and c: arrows) and high diffusion-weighted imaging (DWI) signals (Figure 2b and e: arrows) in right lung base. Concomitant ipsilateral hilar and mediastinal nodes with increased FDG uptake were present (Figure 2a and d: dotted arrows). She had a positive SARS-CoV-2 RT-PCR test and was diagnosed as having COVID-19 pneumonia.^1^ High-resolution CT performed 2 days later showed consolidation in right lung base periphery surrounded by ground-glass opacities (Figure 2f) consistent with COVID-19 pneumonia.^2,3^

## Discussion

Compared with PET/CT, PET/MRI has several strengths such as improved soft tissue contrast and added value of DWI.^4^ On the other hand, limitations of PET/MRI are long scan duration and limited evaluation of pulmonary parenchyma. Although many researchers have recently reported the FDG PET/CT manifestations of COVID-19 infection,^5–7^ to the best of our knowledge, this is the first report showing COVID-19 pneumonia visualized by integrated FDG PET/MRI which simultaneously provides PET and MR functional images. In this case with negative SARS-CoV-2 antigen test and chest X-ray results, incidental high FDG uptake and high DWI signals in the lung aided in the detection of COVID-19 pneumonia. In addition, the use of a metal-free face mask compatible with MRI helped in preventing the dispersal of viral droplets.^8^

SARS-CoV-2 uses angiotensin-converting enzyme-2 (ACE-2) as a functional receptor and high ACE-2 expression is known in alveolar type II cells of the lung and many patients infected with COVID-19 develop pneumonia.^9^ In some cases, the entry of SARS-CoV-2 into alveolar Type II cells and subsequent proinflammatory cytokine release (cytokine storm) may cause acute respiratory distress syndrome. ACE-2 is also found in the heart, small intestine, arterial and venous endothelial cells and smooth muscle cells in organs including the brain.^10^ SARS-CoV-2 infection is a systemic disease that can affect multiple organ systems besides the lungs causing myocardial injury, central nervous system (CNS) and gastrointestinal involvement.^11^ Juengling et al recently pointed novel radiolabeled peptide and antibody PET tracers for evaluating the pathophysiological features of COVID-19 and their relation to current concepts of therapeutical interventions.^12^ Currently established molecular targets possibly suitable for COVID-19 are chemokines and chemokine receptors, ACE-2 and the Type 1 angiotensin-II-receptor (ATR1), post-inflammatory fibrosis, purinergic receptor P2X7, cyclooxigenase-2, and CD-8+ T-lymphocytes etc. Integrated PET/MRI using dedicated tracers could prove to be helpful in cases of occult myositis, endocarditis, myocardial inflammation, or myopathy, as well as CNS involvement. Given the still limited availability of PET/MRI and novel PET tracers and current existing demand for this modality, whole-body PET/MRI could be used for evaluating the pathophysiology of COVID-19 as a systemic disease.

## Learning points

- Incidental high FDG uptake and high DWI signals in the lung aid in the detection of COVID-19 pneumonia.
- The use of an MRI-safe face mask with no metal helps in preventing the dispersal of viral droplets during PET/MRI scans.
- Whole-body PET/MRI could be useful for evaluating the pathophysiology of COVID-19 as a systemic disease.
